# Early Adventitial Activation and Proliferation in a Mouse Model of Arteriovenous Stenosis: Opportunities for Intervention

**DOI:** 10.3390/ijms222212285

**Published:** 2021-11-13

**Authors:** Jenq-Shyong Chan, Yang Wang, Virgilius Cornea, Prabir Roy-Chaudhury, Begoña Campos

**Affiliations:** 1 Division of Nephrology, Department of Internal Medicine, Armed Forces Taoyuan General Hospital, Taoyuan 325, Taiwan; 2 Division of Nephrology, Department of Medicine, Tri-Service General Hospital, National Defense Medical Center, Taipei 114, Taiwan; 3 School of Medicine, National Defense Medical Center, Taipei 114, Taiwan; 4 Division of Nephrology, Department of Internal Medicine, University of Cincinnati, Cincinnati, OH 45267, USA; wyc2168@gmail.com; 5 Department of Pathology, University of Cincinnati, Cincinnati, OH 45267, USA; virgilius_c@yahoo.com; 6 Department of Medicine, Division of Nephrology and Hypertension, University of North Carolina, Chapel Hill, NC 27599, USA; prabir_roy-chaudhury@med.unc.edu; 7 WG (Bill) Hefner Salisbury VA Medical Center, Salisbury, NC 27284, USA

**Keywords:** adventitia, macrophage, proliferation, arteriovenous fistula

## Abstract

Background: Arteriovenous fistula (AVF) stenosis remains an important cause of AVF maturation failure, for which there are currently no effective therapies. We examined the pattern and phenotype of cellular proliferation at different timepoints in a mouse model characterized by a peri-anastomotic AVF stenosis. Methods: Standard immunohistochemical analyses for cellular proliferation and macrophage infiltration were performed at 2, 7 and 14 d on our validated mouse model of AVF stenosis to study the temporal profile, geographical location and cellular phenotype of proliferating and infiltrating cells in this model. Results: Adventitial proliferation and macrophage infiltration (into the adventitia) began at 2 d, peaked at 7 d and then declined over time. Surprisingly, there was minimal macrophage infiltration or proliferation in the neointimal region at either 7 or 14 d, although endothelial cell proliferation increased rapidly between 2 d and 7 d, and peaked at 14 d. Conclusions: Early and rapid macrophage infiltration and cellular proliferation within the adventitia could play an important role in the downstream pathways of both neointimal hyperplasia and inward or outward remodelling.

## 1. Introduction

Arteriovenous fistula (AVF) nonmaturation (defined as an arteriovenous fistula that does not develop an adequate blood flow or lumen diameter to support haemodialysis) is currently a critical clinical problem responsible for significant morbidity, mortality and economic cost [1,2,3]. We have previously demonstrated that AVF nonmaturation is characterized by tight perianastomotic venous narrowing [4], probably due to the combination of a lack of outward (expansive) remodelling and the presence of aggressive venous neointimal hyperplasia [2,5]. AVF stenosis primarily occurs in the peri-anastomotic regions of both radio-cephalic and brachiocephalic AVFs, and also in the cephalic arch region (downstream of brachiocephalic AVFs) [6] Despite the magnitude of the clinical problem, there are currently no effective therapies for AVF nonmaturation. This is likely due to insufficient knowledge about the interactions between haemodynamic injury and the vascular response to injury, especially oxidative stress (likely magnified by the presence of uraemia) [2,5]. We previously developed a mouse model characterized by significant stenosis at the AV anastomosis 14 d post-surgery to better understand the cellular and molecular mechanisms involved in early AVF failure [7,8]. We have also demonstrated rapid and early (2 d) macrophage infiltration in our pig model of AVF stenosis [9].

Therefore, the aim of this study was to delineate the sequential profile and geographical pattern of cellular proliferation and macrophage infiltration at different timepoints in our validated mouse model of AVF stenosis. The results of this study add to the knowledge base at a clinical and patient impact level, aiding the future modulation of biological profiles in AVF maturation, resulting in the identification of novel mechanism-based therapies for the prevention of AVF maturation failure.

## 2. Results

### 2.1. Mouse AVF Model Histology

**Figure 1:**
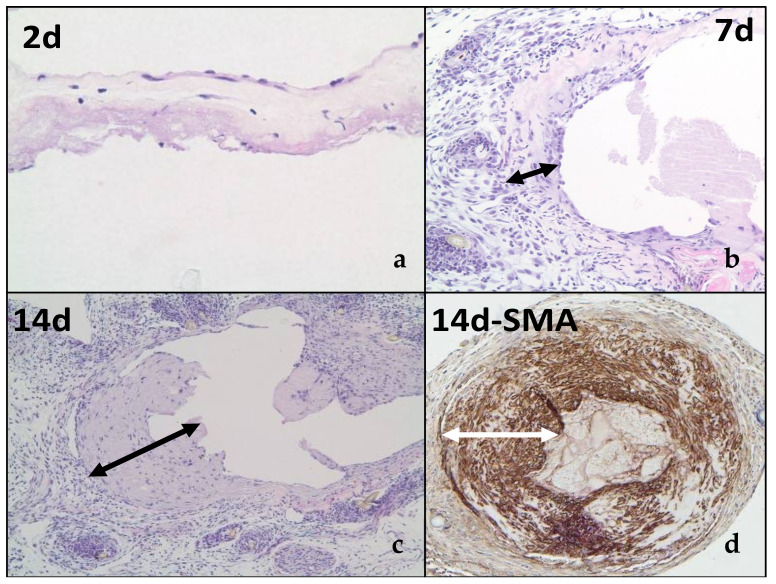
Mouse AVF Model Histology: (**a**–**d**) Note the rapid increase in neointimal hyperplasia from 2 d to 7 d to 14 d. Double-headed arrows in white and black indicate the extent of the neointimal hyperplasia; SMA = smooth muscle alpha actin.

Figure 1 describes the increase in neointimal hyperplasia from day 2 (Figure 1a; no neointimal hyperplasia) to day 7 (Figure 1b; increasing neointimal hyperplasia with increased cellularity) to day 14 (Figure 1c,d; a mature lesion made up primarily of SMA+ve cells (myofibroblasts (synthetic phenotype vascular smooth muscle cells) or contractile phenotype vascular smooth muscle cells). Of note, the findings in Figure 1d are similar to those present in our earlier studies of human AVF maturation failure [10].

### 2.2. Cellular Proliferation

**Figure 2:**
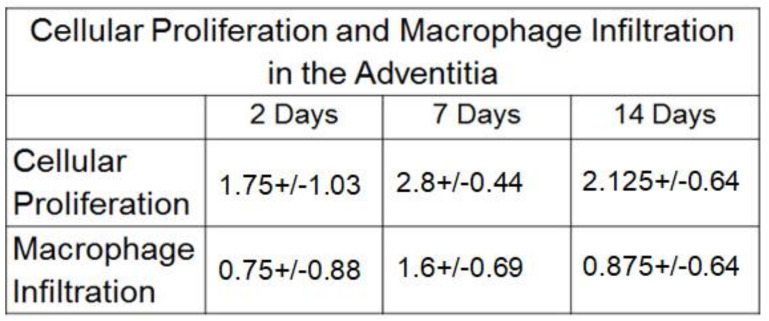
Semi-quantitative scoring for cellular proliferation and macrophage infiltration.

**Figure 3:**
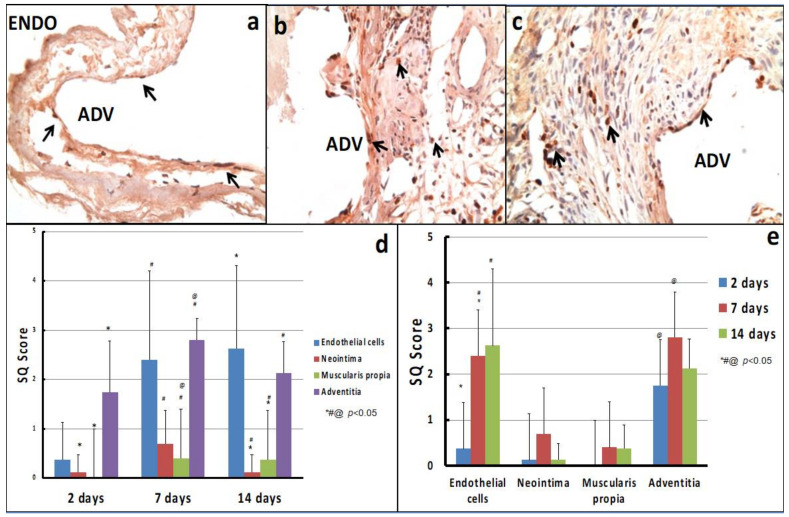
Cellular proliferation in the mouse AVF stenosis model: (**a**–**c**) show the immunohistochemistry for Ki-67 at 2 d, 7 d and 14 d, respectively. Note the early (2 d) and persistent (7 d and 14 d) presence of cellular proliferation in our mouse AVF stenosis model. (**d**,**e**) show our semi-quantitative scoring system for Ki-67 across different time points and different regions of the AV anastomosis. Small black arrows indicate proliferating cells; Endo = luminal side; Adv = adventitial side.

Figure 2 and Figure 3 show active adventitial proliferation as early as 2 d post-AVF creation, which peaks at 7 d and then begins to decline at 14 d. Using our semiquantitative (SQ) scoring scale, the maximal degree of adventitial proliferation at day 7 was 2.8 (an average of nearly 50% of all cells within the adventitia). In contrast, while there was minimal endothelial proliferation at 2 d, this increased before 7 d (SQ score of 2.4) and peaked at 14 d (SQ score of 2.625). Interestingly, there was minimal cellular proliferation in the two histological layers between the adventitia and the endothelium (the media (muscularis propria) and neointimal layers; see Section 3).

### 2.3. Macrophage Infiltration

**Figure 4:**
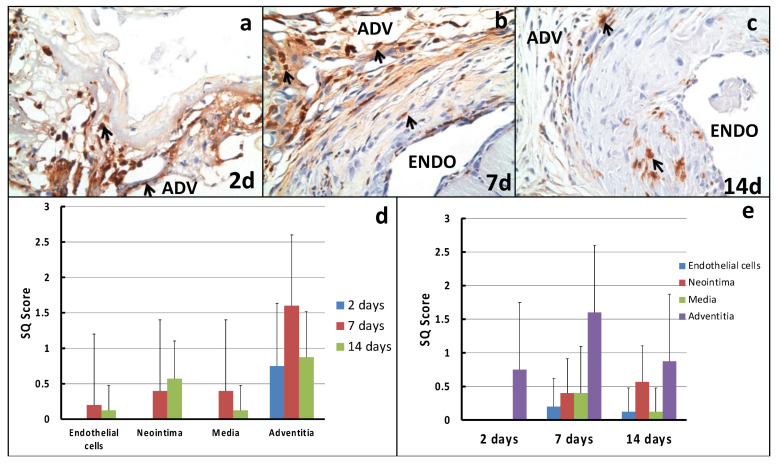
Macrophage infiltration in the mouse AVF stenosis model: (**a**–**c**) show the immunohistochemistry for Mac-2 at 2 d, 7 d and 14 d, respectively. Note the early (2 d adventitia) and persistent (7 d and 14 d) presence of macrophage infiltration in our mouse AVF stenosis model, which is predominantly located in the adventitia but also increases in the neointimal region by 14 d. (**d**,**e**) show our semi-quantitative scoring system for Mac-2 across different timepoints and different regions of the AV anastomosis. Small black arrows indicate macrophages; Endo = luminal side; Adv = adventitial side.

Figure 4 shows early macrophage infiltration at 2 d, which peaks at 7 d (SQ score = 1.6) and then declines to an SQ score of 0.875 at 14 d. Interestingly, a small but steady increase in macrophage infiltration into the neointima peaked at 14 d (lower black arrow in Figure 4c; SQ score of 0.57). While we did, on occasion, identify macrophage infiltration (small granuloma’s) around suture sites, these could easily be differentiated from the predominant macrophage infiltration that was scored.

## 3. Discussion

Our results demonstrate a clear, definite “pattern” of cellular proliferation and macrophage infiltration that occurs within the different layers of the vessel wall at different timepoints in our particular mouse model of AV peri-anastomotic fistula stenosis, as described in the results section. We believe that these results highlight many important mechanistic pathways that determine the pathobiology of peri-anastomotic venous stenosis in our mouse AV fistula model.

The adventitia has historically been regarded as a relatively inert layer acting as a supportive connective tissue and extracellular matrix scaffold around vessels of the vasa vasorum; however, there is now considerable evidence (from experimental models of vascular injury other than AV stenosis) to indicate that, following vascular injury, adventitial cells (fibroblasts and myofibroblasts) migrate and proliferate through the media and into the intima, contributing to the final neointimal volume [11,12].

The data from our studies support this underlying hypothesis that early cellular proliferation and macrophage infiltration within the adventitia likely result in the production of cytokines and matrix metalloproteinases. The former could then diffuse through the media and neointima to result in endothelial cell proliferation, while the latter facilitate cellular migration. However, the lack of active and aggressive cellular proliferation and macrophage infiltration within the media and neointima (intervening layers between the adventitia and endothelium) suggests that secreted mediators play the main role; although the small but definite increase in macrophage infiltration, which peaked in the media at 7 d and at 14 d in the neointima, suggests that some macrophages likely physically migrate from the adventitia into the media and neointima at 7 d and 14 d.

Of note, the relative importance of cytokine-mediated changes versus physical migration of macrophages from the adventitia towards the intima is critical, since this could determine the most effective type of therapeutic intervention for preventing AVF stenosis in this model and for clinical translation. In addition, there are minimal data on whether macrophages and their secreted cytokines enhance or reduce AVF stenosis. However, it is clear that the mediator-release pathway is likely responsible for the initial outward (or inward) remodeling that occurs soon after the creation of an AVF fistula. While our findings support the work of other groups regarding the successful delivery of periadvential therapy in a similar mouse model [13,14,15,16,17], the recent failure of two large, pivotal clinical studies evaluating vonapanitase [18] and the limited efficacy of a sirolimus wrap (effective in patients over the age of 65 only) suggests that the underlying mechanisms might be both more nuanced and more complex, particularly concerning the timeline. We speculate, for example, that we may need to target cellular proliferation and macrophage infiltration at a very specific timepoint. In other words, the overall biological effects of the changes in proliferation and infiltration that we see in our mouse model of AVF stenosis could enhance AVF treatment success at 3 d, but inhibit it at 14 d.

To tease out the potential intricacies of AVF remodelling, we plan to combine a more detailed histological and molecular study with an assessment of real-time luminal narrowing and expansion through the use of MRI/MRA analysis in our mouse model. Interestingly, in our pig models, we have already demonstrated that the venous diameter is smaller than the arterial diameter at 2 d post-surgery, suggesting early inward remodelling.

It is also important to emphasize that the clinical setting could be far more complex than our experimental model, with differences between forearm and upper-arm AVFs (including in the context of pharmacological therapy as shown in the initial vonapanatase studies, where the best results were obtained from forearm AVFs [19] and with a potentially lower dose (personal communication from Steve Burke)), and also in the context of practice patterns across the US, Europe and Japan, as described in detail in recent DOPPS reports [20,21,22].

Additionally, despite our focus on hypothesizing potential pathophysiological pathways for peri-anastomotic AVF stenosis in our mouse model, we are very cognizant of the fact that there could be many differences between our mouse model and the real world clinical setting. We would, therefore, like to make the following brief comments about clinical AVF stenoses in the following settings:

(a) Non-peri-anastomotic stenotic lesions: We speculate that (a) needle stick injuries prior to AVF creation, (b) venous valves and (c) non laminar flow as a result of unique anatomical configurations could be responsible for non-peri-anastomotic clinical stenoses.
(b) Forearm versus upper arm AVFs: In an earlier study by our group that described the cellular composition of neointimal hyperplasia in forearm and upper-arm AVFs, there was no real difference in the cellular phenotype, suggesting that the downstream venous response could be similar regardless of the type of vascular injury or the anatomical location of the same [23].
(c) First time stenosis versus restenotic lesions: While the data on this are limited, we have previously not been able to demonstrate any significant histological changes between these two clinical settings.

In support of the importance of the adventitia in vascular stenoses, we note studies where adventitial injury and activation is associated with vascular stenosis [24,25,26] and also studies by Misra et al. in a mouse AVF stenosis model where adventitial therapy with a blocker of CX3CR1 [27] reduced peri-anastomotic stenosis and adventitial delivery of Vitamin D, both post angioplasty in a mouse model [28], and also reduced venous stenosis. 

In summary, while our data support the importance of the adventitia in AVF maturation in our mouse model of peri-anastomotic AV stenosis, further studies linking molecular and cellular changes at different timepoints to the timeline of venous segment dilatation (outward remodelling) or constriction (inward remodelling), as also the occurrence of neointimal hyperplasia, are urgently needed to translate adventitial therapies that show promise in AVF mouse models into the clinical setting with more success than previously obtained.

## 4. Materials and Methods

### 4.1. Study Design

Fourteen mice were utilized in this validation study, and a total of 14 fistulae were evaluated. Mice were sacrificed at 2 d (4 fistulae), 7 d (5 fistulae), and 14 d (5 fistulae). While these numbers are limited, they were felt to be adequate to identify differences between different timepoints and different vessel wall layers in our mouse model of peri-anastomotic AVF stenosis. 

### 4.2. Arteriovenous Fistula Model Development

**Figure 5:**
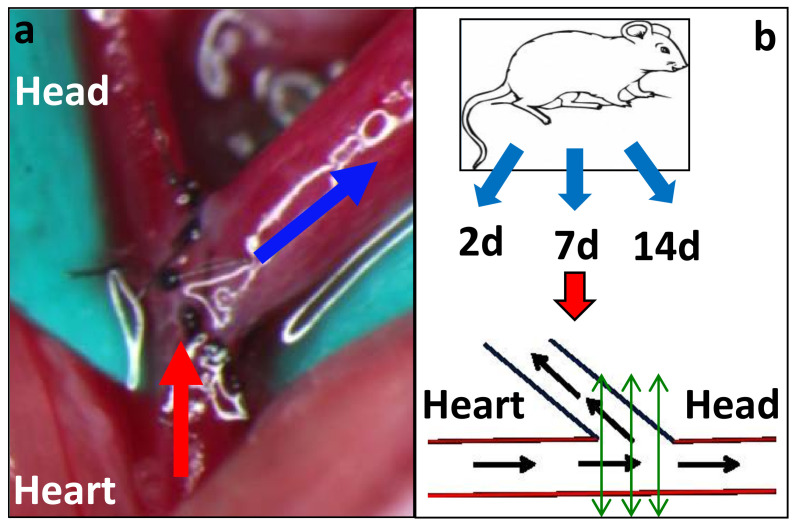
Mouse AVF surgery and histology techniques: (**a**) describes the final AVF between the side of the carotid artery (red arrow) and the end of the jugular vein (blue arrow). (**b**) (lower panel) documents the technique that we will use to section the anastomosis. Black arrows indicate direction of blood flow; green double-headed arrows indicate how we sectioned the anastomotic region. Heart and Head labels help to indicate the directionality of flow in both panels.

Ten-week-old C57BL/6J mice (Jackson Laboratory, Bar Harbor, ME, USA) were anaesthetized, surgically prepped, and administered pre-emptive analgesics. As previously described in a joint paper by us and the Rotmans group, [7,8] AV fistulae were created with an end-to-side anastomosis between the jugular vein and carotid artery (Figure 5) [7,8]. Of note, there was no compression of the vein, and the very fine 11/0 sutures did not cause any luminal obstruction. We also handled the veins and arteries around the anastomosis very carefully to prevent any damage to the adventitia, and though we used clamps, they were vessel clamps that cause minimal trauma. We recognize that there will always be some damage to the vasa vasorum, but this likely mimics the clinical setting and so could contribute to the validity of our model. Intravenous heparin was administered as necessary, and special attention was paid to haemostasis.

We recognize that other surgical techniques have been developed for the creation of mouse AVFs, including the use of a needle to make a connection between the aorta and IVC [29], and also the use of an end of an artery to the side of a vein anastomosis [30]. We strongly believe that this end of a vein to the side of an artery anastomosis is the only one that mimics the clinical setting. 

### 4.3. Specimen Collection and Processing

At the time of sacrifice, the animals were given a lethal injection of sodium pentothal. Animals were sacrificed at 2, 7, and 14 d post-AVF surgery. For the current study, we focused only on AV anastomosis (see green double-headed arrows in Figure 5b).

### 4.4. Processing of Samples for Histology

The explanted fistula was carefully dissected, fixed in formalin for 48 h, coated in paraffin and embedded into a single paraffin block. We then cut the entire block and focused on the anastomotic area, as shown by the green double-headed lines in Figure 5b. We used the section that most closely matched the central green double-headed line in Figure 5b. The chosen section was first assessed with H and E staining (Figure 1).

### 4.5. Immunohistochemistry

Four-micrometer paraffin sections across the anastomotic area from all the AVFs described above at the three different timepoints (2 d, 7 d and 14 d) were assessed for Ki-67, a marker of cellular proliferation (Ki-67; BD Pharmingen, San Diego, CA, USA) and macrophage infiltration (MA-2; Accurate, Carle Place, NY, USA) using the Vectastain ABC Peroxidase mouse-on-mouse kit, with DAB as the peroxidase substrate (Vector Laboratories, Burlingame, CA, USA). All sections were counterstained with Harris’ haematoxylin for 1 min, immersed in saturated lithium carbonate for 1 min, and then mounted in Permount (Fisher, St. Clair Shores, Michigan, USA). Mouse IgG1 was used as a negative control. All sections were examined by one observer (Dr. Cornea) who was blinded to tissue identity, and the location and intensity of the immunostaining were recorded. We used the following semiquantitative scoring scale: 0 = <10% of total cells positive; 1+ = 11–25%; 2+ = 26–50%; 3+ = 51–75%; and 4+ = >75%.

### 4.6. Statistical Analysis

The mean proliferation score for each AV fistula was calculated for each section through the anastomotic region, with separate scores calculated for the adventitia, media, neointima and endothelium at each of the three timepoints for each AVF. The scores for the different AVFs at each timepoint (2 d, 7 d and 14 d) were then averaged, resulting in the data shown in Figure 2, Figure 3 and Figure 4. ANOVA was used to identify differences between the degrees of cellular proliferation and macrophage infiltration within the different layers of the vessel wall at different timepoints. A *p*-value of <0.05 was considered significant.

## Data Availability

All relevant data sets for this publication are available from the corresponding author under reasonable request.
